# C11-hydroxy and C11-oxo C_19_ and C_21_ Steroids: Pre-Receptor Regulation and Interaction with Androgen and Progesterone Steroid Receptors

**DOI:** 10.3390/ijms25010101

**Published:** 2023-12-20

**Authors:** Rachelle Gent, Desmaré Van Rooyen, Stephen L. Atkin, Amanda C. Swart

**Affiliations:** 1 Department of Biochemistry, Stellenbosch University, Stellenbosch 7600, South Africa; rgent@sun.ac.za (R.G.);; 2 School of Postgraduate Studies and Research, Royal College of Surgeons in Ireland Bahrain, Adliya 15503, Bahrain; satkin@rcsi-mub.com; 3 Department of Chemistry and Polymer Science, Stellenbosch University, Stellenbosch 7600, South Africa

**Keywords:** 11β-hydroxysteroid dehydrogenase (HSD11B), 11β-hydroxyandrostenedione (11OHA4), 11β-hydroxyprogesterone (11OHP4), adrenal steroidogenesis, androgen receptor (AR), castration-resistant prostate cancer (CRPC), oxygenated androgens, progesterone receptor (PR)

## Abstract

C11-oxy C_19_ and C11-oxy C_21_ steroids have been identified as novel steroids but their function remains unclear. This study aimed to investigate the pre-receptor regulation of C11-oxy steroids by 11β-hydroxysteroid dehydrogenase (11βHSD) interconversion and potential agonist and antagonist activity associated with the androgen (AR) and progesterone receptors (PRA and PRB). Steroid conversions were investigated in transiently transfected HEK293 cells expressing 11βHSD1 and 11βHSD2, while CV1 cells were utilised for agonist and antagonist assays. The conversion of C11-hydroxy steroids to C11-oxo steroids by 11βHSD2 occurred more readily than the reverse reaction catalysed by 11βHSD1, while the interconversion of C11-oxy C_19_ steroids was more efficient than C11-oxy C_21_ steroids. Furthermore, 11-ketodihydrotestosterone (11KDHT), 11-ketotestosterone (11KT) and 11β-hydroxydihydrotestosterone (11OHDHT) were AR agonists, while only progestogens, 11β-hydroxyprogesterone (11βOHP4), 11β-hydroxydihydroprogesterone (11βOHDHP4), 11α-hydroxyprogesterone (11αOHP4), 11α-hydroxydihydroprogesterone (11αOHDHP4), 11-ketoprogesterone (11KP4), 5α-pregnan-17α-diol-3,11,20-trione (11KPdione) and 21-deoxycortisone (21dE) exhibited antagonist activity. C11-hydroxy C_21_ steroids, 11βOHP4, 11βOHDHP4 and 11αOHP4 exhibited PRA and PRB agonistic activity, while only C11-oxo steroids, 11KP4 and 11-ketoandrostanediol (11K3αdiol) demonstrated PRB agonism. While no steroids antagonised the PRA, 11OHA4, 11β-hydroxytestosterone (11OHT), 11KT and 11KDHT exhibited PRB antagonism. The regulatory role of 11βHSD isozymes impacting receptor activation is clear—C11-oxo androgens exhibit AR agonist activity; only C11-hydroxy progestogens exhibit PRA and PRB agonist activity. Regulation by the downstream metabolites of active C11-oxy steroids at the receptor level is apparent—C11-hydroxy and C11-oxo metabolites antagonize the AR and PRB, progestogens the former, androgens the latter. The findings highlight the intricate interplay between receptors and active as well as “inactive” C11-oxy steroids, suggesting novel regulatory tiers.

## 1. Introduction

The androgen precursor 11β-hydroxyandrostenedione (11OHA4) was first identified to be of adrenal origin in 1953 [1,2] and thereafter, in 1955, was established as a major adrenal metabolite [3]. This steroid attracted a great deal of attention specifically with regards to the metabolic pathways giving rise to this steroid. However, interest declined when investigations into androgenic activity showed that 11OHA4 had low androgenicity compared to testosterone (T) and androstenedione (A4) [4]. It was generally believed that the 11β-hydroxylation of A4 was merely pre-receptor regulation, preventing A4 from being metabolised to T and activating the androgen receptor (AR). It was only in 2011 and 2012 when interest in 11OHA4 was reignited with studies, once again reporting 11OHA4 as a major adrenal C_19_ steroid utilising in vitro models [5]. The “re-discovery” was largely associated with advances in analytical methodologies, specifically, liquid chromatography tandem mass–spectrometry (LC-MS/MS). Steroid analysis was no longer limited by immunoassays which lack specificity. In addition, LC-MS/MS allows for the simultaneous analysis of numerous steroids in a single screening method [5,6,7,8,9]. Subsequent studies have confirmed that 11β-hydroxytestosterone (11OHT), 11-ketotestosterone (11KT) and 11-ketodihyrotestosterone (11KDHT) metabolites resulting from the metabolism of 11OHA4 are AR agonists. This finding confirmed that 11OHA4 was, in fact, not a dead-end product but rather leads to the biosynthesis of metabolically relevant C11-oxy C_19_ steroids which were shown to be of equal androgenicity when compared to T and dihydrotestosterone (DHT), respectively [10].

**Figure 1:**
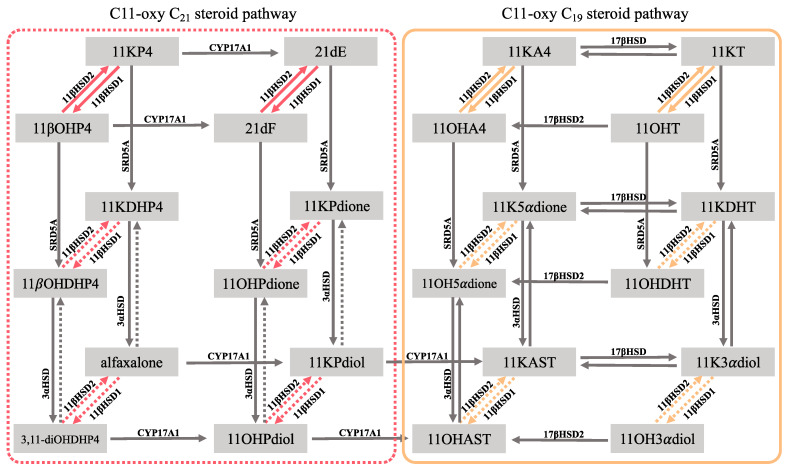
C11-oxy C_21_ backdoor pathway and 11β-hydroxyandrostenedione (11OHA4) pathway. The interconversion of C11-hydroxy and C11-oxo steroids catalysed by 11β-hydroxysteroid dehydrogenase isozymes (11βHSD1/2). C11-oxy C_21_ backdoor pathway, dashed line; 11OHA4 pathway, solid line. **Steroids:** 11βOHP4, 11β-hydroxyprogesterone; 11βOHDHP4, 11β-hydroxydihydroprogesterone; 11KP4, 11-ketoprogesterone; 11KDHP4, 11-ketodihydroprogesterone; 3,11-diOHDHP4, 5α-pregnan-3α,11β-diol-20-one; alfaxalone, 5α-pregnan-3α-ol-11,20-dione; 21dF, 21-deoxycortisol; 21dE, 21-deoxycortisone; 11OHPdione, 5α-pregnan-11β,17α-diol-3,20-dione; 11KPdione, 5α-pregnan-17α-ol-3,11,20-trione; 11OHPdiol, 5α-pregnan-3α,11β,17α-triol-20-one; 11KPdiol, 5α-pregnan-3α,17α-diol-11,20-dione; 11OHA4, 11β-hydroxyandrostenedione; 11KA4, 11-ketoandrostenedione; 11OHT, 11β-hydroxytestosterone; 11KT, 11-ketotestosterone; 11OH5αdione, 5α-androstan-11β-ol-3,17-dione; 11K5αdione, 5α-androstane-3,11,17-trione; 11OHDHT, 11β-hydroxydihydrotestosterone; 11KDHT, 11-ketodihydrotestosterone; 11OHAST, 11β-hydroxyandrosterone; 11KAST, 11-ketoandrosterone; 11OH3αdiol, 11β-hydroxyandrostanediol; 11K3αdiol, 11-ketoandrostanediol. **Enzymes:** 11βHSD1, 11β-hydroxysteroid dehydrogenase 1; 11βHSD2, 11β-hydroxysteroid dehydrogenase 2; CYP17A1, cytochrome P450 17α-hydroxylase/17,20-lyase; SRD5A, 5α-reductase; 3αHSD, 3α-hydroxysteroid dehydrogenase; 17βHSD, 17β-hydroxysteroid dehydrogenase.

Our investigations identified metabolites of the 11OHA4 pathway (Figure 1) as well as the enzymes catalysing its downstream conversion—17β-hydroxysteroid dehydrogenase (17βHSD), 5α-reductase (SRD5A) and 3α-hydroxysteroid dehydrogenase (3αHSD), with 11β-hydroxysteroid dehydrogenase type 2 (11βHSD2) being pivotal [11,12]. 11βHSD isozymes are typically known for the interconversion of cortisone and cortisol with the inactivation of cortisol to cortisone by 11βHSD2 which catalyses the oxidation of the C11 hydroxyl group, thus ensuring selective binding of the mineralocorticoid receptor (MR) by aldosterone [13]. In contrast, 11βHSD type 1 (11βHSD1) reactivates cortisol from cortisone leading to the binding and from the activation of the glucocorticoid receptor (GR) in GR-rich tissues [14]. Data suggest that the 11βHSD isozymes also have a pre-receptor regulatory function regarding the C11-oxy C_19_ steroids. However, conversion by 11βHSD2 yields more active steroids which, in turn, are inactivated by 11βHSD1. This is apparent in the conversions of 11OHT to 11KT and 11β-hydroxydihydrotestosterone (11OHDHT) to 11KDHT, whereby 11KT and 11KDHT have been shown to act as stronger agonists of the AR at 1 and 10 nM when compared to their C11-hydroxy counterparts [5,9], and 11OHT and 11KT are comparable to T at 30 nM [9]. It should, however, be noted that the aforementioned receptor studies were carried out in either MDA-kb2 cells [9] or in COS-1 cells [5]. MDA-kb2 cells express endogenous AR as well as GR [15], while other commonly utilised cell models such as COS-7, HEK293 and Chinese hamster ovary (CHO) cells have been shown to express varying levels of endogenous nuclear steroidogenic receptors—MR and GR in HEK293 and the progesterone receptor (PR), GR and MR in CHO and PR in COS-7 cell models [16,17], potentially resulting in non-specific binding, thus skewing results. An analysis of AR activation in stably transfected CV1 cells showed that the activation by T was 15% higher than 11KT which, in turn, was ~2-fold higher than 11OHT at 300 nM [18]. A subsequent study in the same cell model, which did not express endogenous steroid receptors, reported comparable data at 100 nM; however, induction by 11OHT was markedly lower [19]. Unfortunately, neither 11OHDHT nor 11KDHT or the downstream C11-oxy C_19_ steroid metabolites were included. In our previous receptor assays in HEK293 cells investigating the C11-oxy C_21_ steroids, we showed that 11β-hydroxyprogesterone (11βOHP4) at all concentrations and 21-deoxycortisol (21dF) at 100 nM activated the AR (Figure S1). Another study investigating GR-specific activation using HEK293 cells showed 21dF to have a potency similar to cortisol, while transactivation of the GR by 11βOHP4, 11-deoxycortisol and aldosterone were similar [15].

Similar to the C11-oxy C_19_ steroids, a number of C11-oxy C_21_ steroid metabolites have been reported, also originating from the 11β-hydroxylation of progesterone (P4) and 17α-hydroxyprogesterone (17OHP4) by cytochrome P450 11β-hydroxylase (CYP11B1), leading to the biosynthesis of 11βOHP4 and 21dF, respectively. Both 11βOHP4 and 21dF are also converted to 11-ketoprogestesterone (11KP4) and 21-deoxycortisone (21dE) by 11βHSD2 with the reverse reaction catalysed by 11βHSD1, respectively [20,21]. These steroids are further metabolised (Figure 1), as in the case of the C11-oxy androgens by SRD5A, 3αHSD and cytochrome P450 17α-hydroxylase/17,20-lyase (CYP17A1) [10]. The C11-oxy C_21_ steroids have been identified in circulation across a number of species under physiological and pathophysiological conditions [8,22,23,24,25,26,27,28,29,30]. Although the function of these metabolites remains unexplored, it has been shown that they contribute to the pool of potent C11-oxy androgens in vitro [31,32] and in vivo via CYP17A1 metabolism and downstream conversion by enzymes of the 11OHA4 pathway [29,33,34].

Many disease states have recently been associated with increased levels of C11-oxy C_19_ steroid metabolites such as prostate cancer (PCa), benign prostatic hyperplasia (BPH), polycystic ovary syndrome (PCOS), breast cancer (BC) and endometrial cancer (EC) [23,24,31,35,36,37]. In addition, the expression of both AR and PR isoforms have been identified in tissues (prostate, ovary, breast and endometrium) associated with these pathophysiological conditions [38,39,40,41,42]. As steroid action is mediated via various nuclear steroid receptors, it becomes crucial to determine the nature of these interactions to further understand the role of these C11-oxy C_19_ and C_21_ steroid metabolites under normal and abnormal conditions—which remains largely unexplored.

The aim of this study was, therefore, to investigate pre-receptor regulation by the 11βHSD isozymes in the interconversion of the downstream metabolites of the C11-oxy C_19_ and the C11-oxy C_21_ steroids. Research regarding these downstream metabolites has been hampered due to their commercial unavailability. In addition, downstream reactions catalysed by 11βHSD have, to date, largely been assumed based on steroid analyses in PCa and BPH whole-cell models. The oxidative activity of 11βHSD2 towards the C11-hydroxy C_19_ and C11-hydroxy C_21_ steroid metabolites was therefore determined as well as the reductive activity of 11βHSD1, in the presence of hexose-6-phosphate dehydrogenase (H6PDH), towards their respective C11-oxo C_19_ and C11-oxo C_21_ metabolites in a cell model transiently expressing the 11βHSD isozymes. The agonist and antagonist activity of all the C11-oxy C_19_ and C11-oxy C_21_ steroids and their downstream metabolites towards the AR and the PR isoforms, PRA and PRB, was subsequently investigated in the CV1 cell model.

## 2. Results

### 2.1. The Interconversion of C11-oxy C_19_ and C11-oxy C_21_ Steroids by the 11βHSD Isoforms

#### 2.1.1. Conversion of C11-Hydroxy Steroids by 11βHSD2

**Figure 2:**
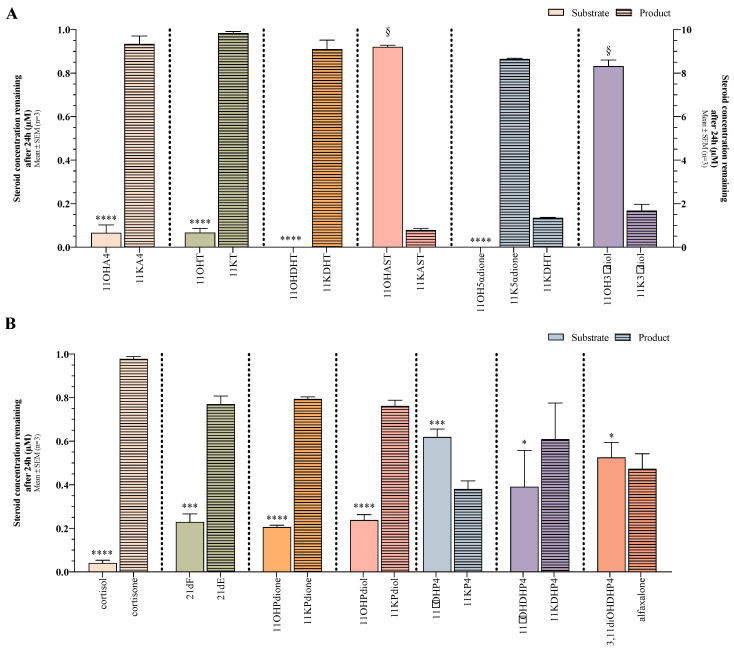
Conversion of C11-hydroxy steroids by 11βHSD2 after 24 h. (**A**) C11-hydroxy C_19_ substrates were added at a concentration of 1 µM, while 10 µM of 11OH3αdiol (shaded area) was added due to inadequate ionization during analysis. Results from this reaction are indicated on the right *y*-axis. (**B**) A total of 1 µM of the substrate was used to evaluate all C11-hydroxy C_21_ steroid conversions. Solid bars, substrate; patterned bars, product. Unpaired *t*-tests were utilised to determine statistical significance between the substrate concentration added (0 h) and the substrate concentration remaining (24 h). §, not significant; *, *p* < 0.05; ***, *p* < 0.001; ****, *p* < 0.0001.

In the 11βHSD2 assays (Figure 2A), the C11-hydroxy C_19_ steroids were efficiently converted to their respective C11-oxo products, with the exception of 11β-hydroxyandrosterone (11OHAST) and 11β-hydroxyandrostanediol (11OH3αdiol) with ~92% and ~83% of the substrate remaining after 24h, respectively. 11OH3αdiol was assayed at a higher concentration due to the low ionisation of the steroid and can, therefore, not be directly compared to the other substrates. Negligible 11OHA4 and 11OHT remained with ~93% of 11OHA4 converted to 11KA4 and ~98% of 11OHT to 11KT. Neither 11OHDHT nor 5α-andostan-11β-ol-3,17-dione (11OH5αdione) were detected with 11OHDHT converted to 11KDHT, while the conversion of 11OH5αdione yielded both 5α-androstane-3,11,17-trione (11K5αdione) and 11KDHT, ~87% and ~13%, respectively.

The conversions of the C11-hydroxy C_21_ steroids (Figure 2B) were overall not as efficient as the C11-oxy C_19_ steroids after 24h. Cortisol, the canonical 11βHSD2 substrate, was converted most efficiently to cortisone (~98%) (Figure 2B). The conversion of 5α-pregnan-11β,17α-diol-3,20-dione (11OHPdione), 5α-pregnan-3α,11β,17α-triol-20-one (11OHPdiol) and 21dF were comparable with ~79% of 11OHPdione converted to 5α-pregnan-17α-diol-3,11,20-trione (11KPdione), ~76% of 11OHPdiol to 5α-pregnan-3α,17α-diol-11,20-dione (11KPdiol) and ~77% of 21dF to 21dE. Meanwhile, ~61% of 11β-hydroxydihydroprogesterone (11βOHDHP4) and ~47% of 5α-pregnan-3α,11β-diol-20-one (3,11diOHP4/11OHallopregnanolone) were converted to 11-ketodihydroprogesterone (11KDHP4) and 5α-pregnan-3α-ol-11,20-dione (alfaxalone/11Kallopregnanolone), and the conversion of 11βOHP4 to 11KP4 was the least efficient with 62% of the substrate remaining after 24 h.

#### 2.1.2. Conversion of C11-oxo Steroids by 11βHSD1

**Figure 3:**
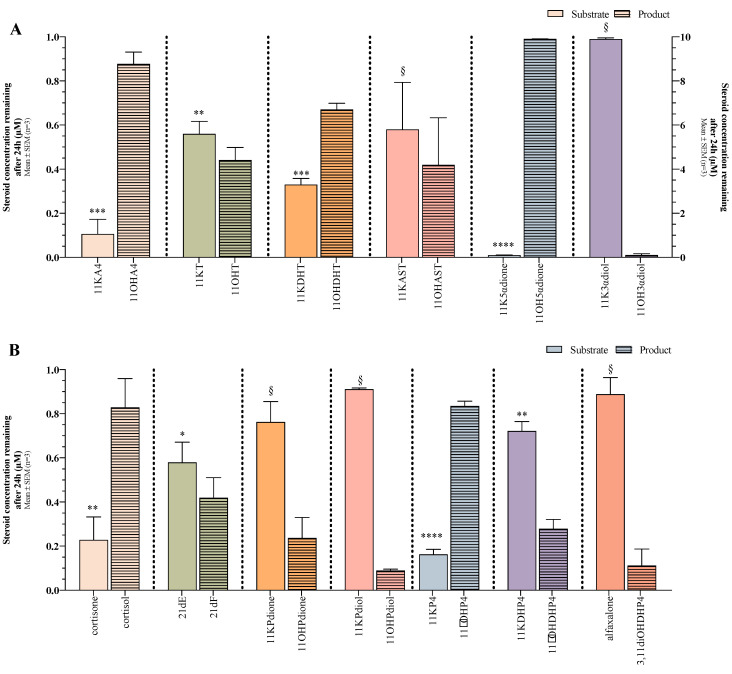
Conversion of C11-oxo steroids by 11βHSD1 after 24h. (**A**) C11-oxo C_19_ substrates were added at a concentration of 1 µM, while 10 µM of 11K3αdiol (shaded area) was added due to inadequate ionization during analysis. Results from this reaction are indicated on the right *y*-axis. (**B**) A total of 1 µM of the substrate was used to evaluate all C11-oxo C_21_ steroid conversions. Solid bars, substrate; patterned bars, product. Unpaired *t*-tests were utilised to determine statistical significance between the substrate concentration added (0 h) and the substrate concentration remaining (24 h). §, not significant; *, *p* < 0.05; **, *p* < 0.005; ***, *p* < 0.001; ****, *p* < 0.0001.

The reverse reactions were subsequently assayed to determine the catalytic activity of 11βHSD1 towards the C11-oxo steroids in the conversion to their respective C11-hydroxy steroids. Overall, the C11-oxo steroids were not converted as readily as the C11-hydroxy substrates in the 11βHSD2 catalysed reactions (Figure 3). The most efficient C11-oxo C_19_ steroid conversion was that of 11K5αdione (~99%) followed by ~88% of 11KA4 converting to 11OHA4 (Figure 3A). The conversion of 11KDHT and 11KT, ~67% and ~44%, respectively, yielded 11OHDHT and 11OHT. Although the data show a conversion of 11-ketoadrosterone (11KAST), the substrate was not significantly lower compared to the initial substrate concentration at T0. The conversion rate of 11-ketoandrostanediol (11K3αdiol) to 11OH3αdiol was similar to the conversion of 11OH3αdiol, with most of the substrate unchanged after 24h possibly being attributed to the higher concentration. This, therefore, does not eliminate a more efficient conversion at lower concentrations.

The conversion of the C11-hydroxy C_21_ steroids by 11βHSD1 (Figure 3B) showed that the catalytic activity towards 11KP4 and cortisone was similar with ~84% of 11KP4 converted to 11βOHP4 and ~83% of cortisone to cortisol. In contrast, ~42% of 21dE was converted to 21dF, while the conversion of 11KDHP4 yielded only ~28% of 11βOHDHP4 and 11KPdione only ~24% of 11OHPdione. Although alfaxalone and 11KPdiol concentrations were lower after 24 h compared to the initial concentrations, the respective products, 3,11diOHDHP4 (~11%) and 11OHPdiol (~9%), nevertheless indicated a conversion by 11βHSD1, albeit a negligible one.

### 2.2. Agonistic and Antagonistic Activity of C11-oxy Steroids towards Nuclear Steroid Receptors

Following the investigation of the catalytic activity of the 11βHSD isozymes, the C_19_ steroids, C_21_ steroids, C11-oxy C_19_ steroids and C11-oxy C_21_ steroids and their downstream metabolites were subsequently screened for ligand potential toward the AR, PRA and PRB for both agonistic and antagonistic responses. The activity of the steroids listed in Table 1 is depicted as a percentage of the highest response elicited by the canonical ligand—1 µM of T and 1 µM of P4 for the AR and PR isoforms, respectively. A non-significant response would, therefore, indicate that the steroid assayed demonstrated similar agonistic activity compared to the canonical ligand, while a significant difference is equivalent to a steroid with higher agonist activity (higher value) or lower agonist activity (lower value) when compared to T or P4. Antagonistic activity is present when a decrease in the T or P4 signal is observed upon treatment. All antagonist responses are depicted as a percentage in comparison to 10 nM of T or P4 without treatment.

#### 2.2.1. Agonistic and Antagonistic Activity towards the AR

Only DHT elicited significantly higher agonist activity compared to T at the lowest concentration, while the response elicited by 11OHDHT, 11KT and 11KDHT were comparable (Table 2A). At 100 nM, the agonist activity of all the steroids increased significantly except for 11KT and 11OHDHT. At 1µM, the agonist activity of DHT and all the pre-receptor C11-oxy androgens was significantly higher (*p*, <0.0001) than that of T.

None of the C11-oxy C_21_ steroids and their metabolites nor any of the downstream C11-oxy C_19_ steroids showed agonist activity towards the AR at any of the concentrations assayed.

Of the C11-oxy C_19_ steroids, only 11OHA4 and 11OH5αdione showed a statistically significant (*p* < 0.05) decrease in T response at 10 µM, albeit lower than that of the known antagonist, bicalutamide (Table 2B). P4, on the other hand, led to the greatest decrease in response at 1 µM followed by 11βOHP4 to a lesser extent. While not statistically significant, 1 µM of 11βOHDHP4, 11KP4, 11KDHP4 and 17OHP4 showed a decrease in T response comparable to that of bicalutamide. At 10 µM, C11-hydroxy C_21_ steroids 11αOHP4, 11αOHDHP4 and 11βOHDHP4 showed a decrease in the elicited T response and were demonstrated as weaker antagonists compared to bicalutamide, while 17OHP4 as an antagonist was comparable. P4 and 11βOHP4 showed the greatest antagonistic activity even when compared to the canonical antagonistic ligand. Neither bicalutamide nor any of the steroids assayed exhibited antagonistic effects at 10 or 100 nM (Table S1).

#### 2.2.2. Agonistic and Antagonistic Activity towards the PRA

P4 was consistently the most active steroid at all concentrations. 17OHP4 and the C11-oxy C_21_ steroids, 11βOHP4, 11αOHP4 and 11βOHDHP4 exhibited agonist activity at all concentrations assayed, although it was lower than that exhibited by P4. While all the listed steroids were able to significantly activate the receptor, it was only 11βOHP4 at 1 µM that induced a response comparable to P4 (Table 3). None of the C_19_ steroids or the C11-oxy C_19_ steroids elicited a response towards the PRA.

None of the steroids assayed exhibited antagonism of the PRA in the presence of 10 nM of P4 (Table S2) besides the natural ligand, RU486, which led to a significant decrease in P4 response.

#### 2.2.3. Agonistic and Antagonist Activity towards the PRB

At 10 nM, the C11-oxo androgen, 11K3αdiol, showed agonist activity comparable to P4. Albeit to a lesser degree, the C11-hydroxy progestogen, 11βOHP4, also showed a statistically comparable increase in signal. Moreover, 11βOHP4 elicited the greatest response at 100 nM and 1 µM in comparison to P4. The 5α-reduced and C11-keto metabolites, 11βOHDHP4 and 11KP4, functioned only as partial agonists together with 11αOHP4 across all concentrations, similar to 17OHP4 (Table 4A).

Only the C_19_ and C11-oxy C_19_ steroids acted as antagonists of the PRB (Table 4B). A4 and its downstream C11-hydroxy metabolite, 11OHA4, showed comparable activity across all concentrations and were far more effective in decreasing the signal elicited by P4 in comparison to RU486 at 10 nM. Similarly, 11OHT and 11KT led to a statistically significant signal reduction, albeit to a lesser extent, while 5α-androstanedione (5αdione) and DHT both only showed partial antagonistic activity. At 100 nM, RU486 demonstrated strong antagonistic activity, while none of the steroids assayed showed comparable activity. A4 and 11OHA4 did, however, exhibit the greatest reduction in P4 response of the C_19_ and C11-oxy C_19_ ligands. Moreover, T and its downstream metabolites, DHT and 11OHT, had comparable responses at 100 nM. Dissimilar to the observed trend, A4 and T led to the greatest decrease in the resulting P4 response at 1 µM, while 11OHA4 and DHT demonstrated comparable outcomes. Lastly, at 10 µM, all listed steroids led to a reduction in the P4 signal and were shown to only be partial antagonists of the PRB in comparison to the canonical antagonist, RU486.

## 3. Discussion

Investigations into the metabolism of the C11-oxy C_19_ and C11-oxy C_21_ steroids have suggested additional regulatory roles for 11βHSD regarding receptor interaction, as has been shown with the C11-oxy C_19_ steroids and the AR. The first step was to identify 11βHSD substrates, followed by determining whether or not these steroids may act as AR or PR ligands.

The 11βHSD isozymes are linked to pre-receptor regulation particularly in the case of the interconversion of cortisol and cortisone, ensuring the specific binding of aldosterone to the MR. The MR has broad substrate specificity and binds 11-deoxycortisol, corticosterone (CORT), cortisol and P4 with affinities similar to that of the canonical ligand, aldosterone (K_d_ = 0.5–3 nM). On the other hand, the GR is more selective, demonstrating a high affinity towards cortisol and CORT (K_d_ = 20–70 nM) [43,44,45]. While aldosterone binds only to MR, cortisol binds both the MR and, with a lower binding affinity, the GR. Since cortisol circulates at higher concentrations than aldosterone, cortisol’s affinity for the MR must be rigorously regulated. Co-expression of the MR and 11βHSD2 avoids non-specific binding with the conversion of cortisol to cortisone, which is unable to bind the MR or the GR, thus ensuring ligand-binding specificity and the subsequent activation of the GR by cortisol. In addition, although aldosterone contains a C11-hydroxyl group, it is not a substrate for 11βHSD2 due to cyclisation with the C18 aldehyde forming an 11,18-hemiacetal group. Due to this unique structural entity, aldosterone, in its hemiacetal form, does not compete with cortisol for binding to 11βHSD2 and remains free to bind the MR [46]. Furthermore, the tissue-specific expression of receptors and 11βHSD isozymes also ensures specificity, as is observed with the MR with its expression generally restricted to aldosterone target tissues such as the kidneys, colon and salivary glands [47]. 

The enzymatic assays were carried out in the HEK293 cell model since these are mammalian in nature and the genomics and transcriptomics of these cells closely resemble that of adrenal cells [48,49,50]. The cell model is, therefore, suitable for investigations into specific enzymatic assays and steroidogenic conversions together with the fact that they express little to no adrenal steroidogenic enzymes due to being immature cells. Although classed as non-steroidogenic, previous studies have shown the endogenous expression of some enzymes in the HEK293 cell line. While not observed in the present study, one such enzyme includes SRD5A which catalyses the reduction of the C4/C5 double bond [17,51,52]. However, conversion of 11OH5αdione by 11βHSD2 in the present study yielded both 11K5αdione and 11KDHT, suggesting the expression of endogenous 17βHSD isozyme/s, which was also reported in a previous study by our group [32]. Past studies investigating enzyme expression in cell models have not identified a known 17βHSD isozyme in HEK293 cells [17]. A novel enzyme, androgen-regulated (prostate) short-chain dehydrogenase/reductase 1 (PSDR1), has, however, been identified [17]. It has been postulated that PSDR1 relates to 17βHSD activity due to the high level of homology demonstrated between PSDR1 and several 17βHSD isozymes [53,54]. Nonetheless, endogenous enzymes account for less than 6% conversion in transfected cells and thus do not significantly impact transfection-based studies and, therefore, are better suited to these investigations [55]. Therefore, although we previously demonstrated 11βHSD conversions in whole-cell models, assays in transiently transfected HEK293 cells allow for investigations into specific intermediate reactions. Direct comparisons between substrates and products are possible due to a reduction in interference or a competition of endogenous steroidogenic enzymes in comparison to whole-cell models expressing endogenous steroidogenic enzymes. 

The C11-hydroxy steroids, 11OHA4, 11OHT and 11OHDHT, were more readily converted by 11βHSD2 when compared to the conversion of the C11-oxo steroids by 11βHSD1. Moreover, data show the conversion of C11-oxo C_19_ steroids to be more efficient when compared to those of C11-oxy C_21_ steroids. These trends have previously been observed when comparing the kinetic parameters, apparent K_m_ and V_max_ values, of some of these reactions (11OHA4, 11OHT, 11βOHP4 and their respective C11-oxo derivatives) together with the canonical steroids, cortisol and cortisone [11,56,57]. Data also show that 11OHAST is a poor 11βHSD2 substrate with more than 80% of it not converted to 11KAST. Although investigations into inactive downstream C11-oxy C_19_ steroids are limited, 11OHAST in serum has been reported to be consistently higher than 11KAST, corroborating our in vitro findings [35,58,59,60]. In both men (21–72 yrs) and women (premenopausal), circulating 11OHAST was significantly higher than 11KAST (~100-fold) [60]. We have also found that 11OHAST is also markedly higher than 11KAST in PCOS patients (unpublished data). While neither 11OHAST nor 11KAST appear to be ideal substrates for the 11βHSD isoforms, both convert 11OH5αdione and 11K5αdione very efficiently. Detected in vivo concentrations of 11OHAST and 11KAST may, therefore, originate from the downstream conversion of 11OH5αdione and/or 11K5αdione by 3αHSD or from the C11-oxy C_21_ steroids [20]. It is, however, important to note that single conversions, as those observed in this study, are only indicative of conversion efficiency, while kinetic parameters are required to make conclusions regarding catalytic efficiency.

Considering the C11-oxy C_21_ steroids, 21dF and its downstream metabolites 11OHPdione and 11OHPdiol were readily converted to their C11-oxo forms by 11βHSD2 (~80%), while the conversion of C11-oxo to the C11-hydroxyl forms was poor with 60–90% of the substrates remaining as C11-oxo C_21_ steroids. The data, therefore, suggest that these C11-oxo C_21_ steroids would be the predominant metabolites, which is of particular relevance in congenital adrenal hyperplasia (CAH) caused by mutations in cytochrome P450 21A2 (CYP21A2), resulting in 21-hydroxylase deficiency (21OHD). A major metabolic pathway, identified in CAH-diagnosed neonates, reported the hydroxylation at C6 of the 3α,5β-reduced urinary metabolite of 21dE. In this study, the C11-oxo metabolites were 2-fold higher than the C11-hydroxyl metabolites. The authors concluded that metabolites of 21dE are potentially more reliable markers of CAH [61]. In CAH, currently, 17OHP4 serves as a marker for circulating levels, as this steroid is increased due to its impeded conversion to 11-deoxycortisol. Although it has been suggested that 21dF should replace 17OHP4 as a marker in diagnostic testing [62], it would be prudent to include 21dE, perhaps more so. 

Earlier reports have shown that 21dF concentrations are increased in CAH [25,63,64,65]. Not included in diagnostic testing, as in the case of 21dE, is 11βOHP4, which is also elevated [26,27,65]. Limited investigations have included 11βOHP4 and 11KP4 in the analysis of CAH steroid profiles [61,66,67]. Although it is widely reported that 11βOHP4 is an inhibitor of 11βHSD2 [68], we have shown that the enzyme does catalyse the conversion of 11βOHP4 to 11KP4. However, the conversion efficiency of 11βOHP4 and its metabolites is low at 40–60% conversion, which is far lower than the conversion of 21dF and its downstream metabolites. While neither 11KDHP4 nor alfaxalone were readily converted by 11βHSD1, 11KP4 was an excellent substrate, suggesting that circulating 11βOHP4 would probably be higher than 11KP4. This is, of course, dependent on the peripheral downstream conversion by SRD5A and 3αHSD and the subsequent clearance.

Pre-receptor regulation is attributed to the conversion efficiency of these 11βHSD-catalysed reactions in vivo and relies on peripheral concentrations of these steroid metabolites to ensure ligand availability for receptor binding. Circulating steroid metabolite concentrations are often measured in various studies to indicate physiological relevance but are not indicative of tissue concentrations or of tissue distribution throughout the body. Several studies indicate a higher intra-tissue concentration of metabolites in comparison to circulating levels, such as the ~1500-fold higher P4 in endometrial biopsies versus serum ones [69] and the presence of 11KA4 and 11OHT in prostate tissue while they are not detected in plasma [36]. Moreover, this has been more easily investigated in animal models which show a similar scenario in murine tissues [70,71]. Peripheral metabolism may, therefore, increase the pool of intra-tissue steroid metabolites as well as elevated active steroids with the potential to bind receptors in various target tissues. The conversion efficiency demonstrated in this study and thus the regulatory consequence of the 11βHSD isoforms would, therefore, depend on the target tissue, downstream steroidogenic enzymes and the level at which they are expressed together with available cofactors.

Active steroids elicit their associated responses in target tissues by binding nuclear steroid receptors, leading to genomic consequences. Two groups of receptors were of interest in the current study, the AR and PR, respectively. Our group previously showed the transactivation of the AR by T, 11KA4, 11OHT, 11KT, 11OHDHT and 11KDHT as well as 11OH5αdione, 11K5αdione, even at low concentrations [12]. Investigations into receptor interactions undertaken in cell models expressing endogenous steroid receptors yield conflicting data, as previously mentioned, which was also apparent in our investigations into HEK293 cells (Figure S1). At all concentrations assayed, 11βOHP4 activated the PRA and was comparable to P4, while at 10 nM, activation of the PRB was 2.5-fold higher than P4, with 21dF being 1.8-fold higher. The PRB was activated by 1 nM of dihydroprogesterone (DHP4), while 11βOHP4 and 11KDHP4 exhibited agonist activity towards the PRB, comparable to P4. These data are in contrast to findings in the current model stably expressing the AR and luciferase genes which presented optimal conditions, eliminating variable expression and interference from endogenous receptors.

The results show DHT to elicit the most significant agonistic response with the AR at lower concentrations, aligning with previous studies highlighting DHT as a potent androgen [18]. Nevertheless, C11-oxy C_19_ metabolites may have a greater impact in vivo, specifically within target tissues. In BPH and castration-resistant prostate cancer (CRPC), the relative contribution of the C11-oxy C_19_ steroids to the androgen profile detected in tissue far outweigh that of the canonical C_19_ steroids. Moreover, 11KDHT is also present at a greater concentration than DHT and T, while 11KT and 11OHDHT are also detected [23,58]. SRD5A isozymes have been identified in the above pathophysiology [72], catalysing the conversion of 11OHT and 11KT to the more potent 5α-reduced metabolites, 11OHDHT and 11KDHT. In addition, 11βHSD2 has also been identified in the absence of 11βHSD1 [73]. Taken together and given the greater efficiency of the 11OHT and 11OHDHT conversion to 11KT and 11KDHT, the flux through the pathway is likely to be towards that of the active C11-oxy androgens in conditions such as CRPC. In contrast, C11-oxy C_21_ steroids are more so associated with antagonism of the AR. The data also demonstrate the inactivation of both AR agonists and antagonists by 3αHSD, associating this steroidogenic enzyme with the overall inactivation of C11-oxy steroids. Although these steroids may not lead to a response on their own, their combined effect should be taken into account. The C11-oxo group of 11KT and 11KDHT is suggested to hamper binding to UGTs, leading to inefficient inactivation by C17 glucuronidation [58,74]. These C11-oxo steroids would, therefore, likely remain active for longer periods when compared to the canonical androgenic ligands. 

Our data show that while the canonical steroid P4 is the more potent agonist for both PRA and PRB at low concentrations, 11βOHP4 elicits a PRA response comparable to P4 and is the superior agonist toward the PRB at higher concentrations. Although high concentrations are required to elicit a response, 11βOHP4 is not readily converted to 11KP4 by 11βHSD2, which could lead to increased tissue concentrations or a downstream metabolism via SRD5A [20] to produce the PR agonist 11βOHDHP4. This could be of relevance in disease states such as BC where the expression of SRD5A isozymes is upregulated [75,76]. Moreover, 11αOHP4 was also an agonist of the PRA and PRB. Although only a difference in the configuration of the C11-hydroxyl group, the function of 11αOHP4 seems to differ. Both steroids are believed to be inhibitors of 11βHSD2; however, the conversion of 11βOHP4 (~40%) has still been demonstrated, while both steroids are also SRD5A substrates [20,31]. Although the origin, role and function of 11αOHP4 remain to be elucidated, the present data do, however, show that a β-hydroxylated C11 group would impart greater PR agonistic activity to the cohort of C_21_ steroids. Furthermore, as 11KP4 does not act as an agonistic ligand of the PRA and is only a partial PRB agonist, it can be concluded that, in contrast to the C11-oxy androgens, 11βHSD1 is pivotal in the activation of C11-oxo C_21_ steroids to C11-hydroxy C_21_ steroids. Moreover, the conversion of 11βOHP4 by SRD5A to 11βOHDHP4 reduces PR-agonist activity. Despite this, 11βOHDHP4 still presents with activity greater than 11αOHP4 towards both PRA and PRB, further emphasising the role of the C11-oxy moiety in imparting the agonistic activity of these steroids. In addition, 11K3αdiol was identified as a PRB agonist at 100 nM and 1 µM. Although circulating concentrations have yet to be established, a cohort of precursor steroids have, however, been detected [35,77]. 11K5⍺diol is a direct product resulting in the biosynthesis of 11KDHT or 11KAST by various AKR1C/17βHSD isozymes [77]. These enzymes are distributed across various tissues but are specifically present in female reproductive tissues [76]. In addition, although a small sample size, the expression of these enzymes is enhanced in female reproductive carcinomas consisting of BC, cervical, EC and ovarian cancer [78]. The PRB is expressed in these tissues and is sometimes upregulated depending on the cell type, prognostic outcome and hormone responsiveness of the cancer [79,80]. 11K3αdiol may very well act as a physiological agonist of this receptor in vivo, even at low concentrations equivalent to P4.

Ligands antagonising canonical steroid ligands were also identified. C11-hydroxy C_19_ steroids, 11OHA4 and 11OH5αdione demonstrated only partial antagonistic activity at 10 µM. Neither steroid has to date been identified as such. Notably, circulating concentrations of 11OHA4 are reported as 0.58–9.47 nM, while that of 11OH5αdione has yet to be established [77]. The data show that both 11OHA4 and 11OH5αdione are efficiently converted by 11βHSD2, at ~98% and ~100%, respectively. It is, therefore, likely that in peripheral target tissues where 11βHSD2 is expressed, these metabolites are more likely to contribute to the active androgen pool. In addition, P4 was demonstrated to be the best antagonist of the AR, aligning with previous reports having identified P4 as an important regulator of AR expression [81]. Moreover, 11βOHP4 was shown to be a better AR antagonist than P4 and bicalutamide, the nonsteroidal AR antagonist generally used in PCa treatment. As 11βOHP4 is not efficiently metabolised by 11βHSD2, flux through the pathway would likely shift to the biosynthesis of 5α-reduced products. A decrease in AR antagonistic activity was associated with the reduction of 11βOHP4 and 11KP4 to 11βOHDHP4 and 11KDHP4, while the conversion of 21dE to 11KPdione, implicating the C17-hydroxyl group, led to increased antagonistic activity. In conditions such as 21OHD, in the absence of CYP21A2, P4 metabolism is shunted towards C11-oxy C_21_ steroid biosynthesis, by the conversion to 11βOHP4 by CYP11B.

It is possible for the C11-oxy C_21_ steroids to contribute to active C11-oxy C_19_ steroids, leading to the activation of the AR, specifically in pathophysiologies characterized by hyperandrogenism. Our in vitro studies have shown the production of 11KDHT in the C11-oxy C_21_ backdoor pathway. In addition, in vivo studies have demonstrated that while the administration of radiolabelled 21dF to CAH patients resulted in the production of 3α, 5α-reduced metabolites of 21dE and 21dF, the C11-oxy C_19_ steroid metabolites, 11OHAST, 11KAST, 11-hydroxyetiocholanolone and 11-ketoetiocholanolone were also identified as urinary metabolites [29,34]. It is apparent that while the C11-oxy C_21_ backdoor pathway may contribute to 11KDHT levels in disease states, the steroid intermediates within the pathway may also exacerbate conditions activating the AR directly. Studies have furthermore shown that the mutated AR, typical of these conditions, becomes sensitised and less specific and can be trans-activated by antagonists such as P4 [82]. The increased induction of mutated AR variants by P4, 11βOHP4, 17OHP4 and 21dF when compared to wild-type AR showed that the latter two steroids generated signals greater than DHT [83]. It is, therefore, likely that, although identified as antagonists, these C11-oxy C_21_ steroids, relevant in disease states, could function as either agonists of AR mutants or could contribute to active androgen biosynthesis. No steroids were identified to significantly reduce the PRA signal, while only C_19_ and C11-oxy C_19_ steroids presented as antagonists of the PRB. The PR isoforms are mainly expressed together with the AR in the breast, endometrium, fallopian tubes and cervix [78]. While steroids were only able to partially antagonise the PRB, the antagonistic activity at 10 nM was greater than the canonical antagonist, RU486. A regulatory role for 11βHSD2 is indicated with the enzyme, reducing the antagonistic activity of the C11-oxy C_19_ steroids due to 11OHA4 and 11OHT showing a greater decrease in signal at 10 nM than their C11-oxo counterparts. As both conversions catalysed by 11βHSD2 are efficient at these low concentrations, it is likely that although this subset of steroids would antagonise the PRB, they would also activate the AR. This may be of relevance in BC and EC, in which several studies have demonstrated the complex relationship between the oestrogen receptor, PR, and AR. Unfortunately, the role and function of androgens in breast and endometrium cancer has not been fully elucidated [84,85].

In conclusion, this study has identified the potential contribution of the 11βHSD isoforms in pre-receptor regulation, as metabolised C11-oxy steroids present as agonistic and antagonistic ligands of the AR and PR isoforms. The data demonstrate the change in ligand activity upon conversion by 11βHSD while also indicating the role of SRD5A. To summarise, firstly, 11βHSD2 increased C11-oxy C_19_ AR agonistic activity but had a contrasting function of reducing the antagonistic activity of progestogens toward the AR. Secondly, in contrast, the conversion of C11-oxy C_21_ steroids by 11βHSD2 leads to a reduced agonistic interaction with the PRA and PRB. SRD5A reduces the activity of both agonists and antagonists across receptors. Lastly, 3αHSD would lead to the overall inactivation of the steroid metabolites except for 11K3αdiol which would present with PRB agonism at low concentrations. Although unclear as to the function of the steroid/receptor complexes, the present study highlights the associated regulatory function of steroidogenic enzymes, not only SRD5A and 3αHSD but also, more specifically, the 11βHSD isoforms.

## 4. Materials and Methods

### 4.1. Materials

A4, T, 5αdione, DHT, AST, P4 and bicalutamide were purchased from Sigma Aldrich (St. Louis, MO, USA) while 11OHDHT, 11OH3αdiol, 11K3αdiol, 11βOHDHP4, 3,11-diOHDHP4, 11OHPdione, 11OHPdiol and 11KPdiol were obtained from IsoScience (Ambler, PA, USA). Moreover, 11OHA4, 11KA4, 11OHT, 11KT, 11OH5αdione, 11K5αdione, 11KDHT, 11OHAST, 11KAST, 11αOHP4, 11αOHDHP4, 11βOHP4, 11KP4, 11KDHP4, alfaxalone, 21dF, 21dE and 11KPdione were purchased from Steraloids (Newport, RI, USA). Deuterated steroids, progesterone 2,2,4,6,6,17,21,21,21-D9 (D9-P4), 17α-hydroxyprogesterone 2,2,4,6,6,21,21,21-D8 (D8-17OHP4), 21-deoxycortisol2,2,4,6,6,21,21,21-D8 (D8-21dF) and 4-androsten-11β-ol-3,17-dione 2,2,4,6,6,16,16-D7 (D7-11OHA4) were obtained from Cambridge Isotopes (Andover, MA, USA). Furthermore, deuterated 11-ketodihydrotestosterone 16,16,17A-D3 (D3-11KDHT) and 11-ketotestosterone 16,16,17A-D3 (D3-11KT) were acquired from Cayman Chemical Company (Ann Arbor, MI, USA). Notably, all steroids used had a minimum of 98% purity. Cell growth media, Dulbecco’s Modified Eagle’s Medium (DMEM) and DMEM/F12 and geneticin (G418) together with hygromycin were acquired from Sigma Aldrich (St. Louis, USA). Moreover, the foetal bovine serum (FBS), penicillin streptomycin and trypsin-EDTA were purchased from Thermo Fisher Scientific (Waltham, MA, USA). All Corning® CellBIND® Surface culture flasks and plates were purchased directly from Corning Life Sciences® (New York, NY, USA) and the XtremeGene HP^®^ DNA transfection reagent from Roche Diagnostics (Manheim, Germany). Lastly, Countess® cell counting chamber slides and a trypan blue stain solution (0.4%) was obtained from Invitrogen (Eugene, OR, USA), while coelenterazine was purchased from Gold Bio (St. Loius, MO, USA). 

#### Cell Models and Vector Constructs

HEK293 cells were purchased from American Type Culture Collection (Manassas, VI, USA) and cultured in DMEM. CV1 monkey kidney cell lines, stably transduced with the *Guassia Luciferase* gene alone (CV1-luc) and with both the *Gaussia Luciferase* and human AR genes (CV1-ARluc), were obtained from Dr William E. Rainey (University of Michigan, USA) and were grown in DMEM/F12 [18]. Growth media for all cell lines were supplemented with NaHCO_3_ (1.5 g/L), FBS (10% *v*/*v*) and penicillin-streptomycin (1% *v*/*v*), and cells were further incubated in a controlled environment at 37℃, 5% CO_2_ and 90% relative humidity. Moreover, CV1-luc cells required the addition of G418 (1.2 mg/mL), while the growth media for CV1-ARluc cells were supplemented with both G418 (1.2 mg/mL) and hygromycin (0.15 mg/mL) to ensure the selection of stably transduced cells. All cell lines were cryogenically stored prior to use and were tested to ensure the absence of mycoplasm. Experimental procedures were carried out once cells reached no more than 80% confluency and a minimum of three passages.

For the steroidogenic assays, the 11βHSD1/pCR3, 11βHSD2/pCR3 and H6PDH/pcDNA3.2 plasmid constructs were purchased from Prof P Stewart (University of Leeds, Leeds, UK). pCIneo was used as a negative control due to the absence of a cDNA insert and was purchased from Promega (Madison, WI, USA). In addition, PR-A/pcDNA3.1 (accession: NM_001202474.3) and PR-B/pcDNA3.1 (accession: NM_000926.4) were custom synthesized and purchased from GenScript (Piscataway, NJ, USA) and were transformed upon receival for use in the receptor assays using the Inoue method [86]. Briefly, JM109 cells were made competent and subsequently transformed with the respective plasmids in parallel to the transformation of puc18 as the control. The ZymoPURE^TM^ II Plasmid Maxiprep Kit was purchased from Inqaba Biotec^TM^ (Menlo Park, South Africa) and was utilised to prepare and purify all plasmid DNA, as per the manufacturers’ instructions. Purified cDNA sequences were verified at the DNA Sequencing Unit situated at the Central Analytical Facility at Stellenbosch University (Stellenbosch, South Africa) and were stored at −20 °C prior to use. 

### 4.2. 11βHSD Steroidogenic Assays

#### 4.2.1. Substrate Addition

Confluent HEK293 cells were seeded into Corning^®^ CellBIND^®^ surface 24-well plates at a cell count of 2 × 10^5^ cells/mL (500 µL/well) and were transiently co-transfected with 0.25 µg/well of 11βHSD1/pCR3 and 0.25 µg/well of H6PDH or 0.25 µg/well of 11βHSD2/pCR3 and 0.25 µg/well of pCIneo. In addition, cells transiently transfected with 0.50 µg/well of pCIneo only served as a negative control for all conversions. All transfections were carried out using the XtremeGene HP^®^ transfection reagent, as per the manufacturer’s instructions. Subsequently, all transfected cells were incubated for 48 h, after which media were removed and replaced with fresh growth media containing either 1 µM (11OHA4, 11KA4, 11OHT, 11KT, 11OH5αdione, 11K5αdione, 11OHDHT, 11KDHT, 11OHAST, 11KAST, 11βOHP4, 11KP4, 21dF, 21dE, 11OHDHP4, 11KDHP4, 11KPdiol, 11KPdione, 3,11diOHDHP4, alfaxalone, 11OHPdiol, 11OHPdione, cortisone and cortisol) or 10 µM (11OH3αdiol and 11K3αdiol) where metabolites would not ionise effectively during the mass spectrometry analysis. Moreover, a substrate was added to the wells absent of cells which were to be used as initial timepoints to confirm the concentration of steroids added to the cells. Thereafter, all plates were incubated for a further 24 h, after which sample aliquots (500 µL) were collected and a mix of deuterated internal steroid standards (100 µL/sample) were added. Deuterated steroid standards included 1 ng of D8-21dF, 1.5 ng of D7-11OHA4, 10 ng of D9-P4 and D8-17OHP4 and 5 ng of D3-11KDHT and D3-11KT. Samples were stored at 4 °C prior to steroid extraction.

#### 4.2.2. Steroid Extraction

A liquid–liquid extraction method was carried out in order to extract and concentrate all steroid metabolites of interest from the media collected from cells [36]. Samples were vortexed and frozen at −80 °C after the addition of MTBE (1.5 mL/500 µL of the sample). Subsequently, the liquid organic phase was collected and dried under a stream of nitrogen at 50 °C, after which the steroids were resuspended in 50% HPLC-grade methanol (150 µL). Resuspended samples were carried over to appropriate vials and stored at −20 °C prior to analysis.

#### 4.2.3. Separation and Quantification by UPC^2^-MS/MS 

Steroid standards (1 mg/mL) in 100% ethanol were used to prepare a range of stock solutions (0.1, 10, 1000, 2000 and 5000 ng/mL) in 100% methanol for use in the standard series used for the quantification of identified metabolites. A standard dilution series ranging from 0.001 ng/mL to 2000 ng/mL was prepared in DMEM (500 µL), and steroids were extracted in parallel to the samples, as described in Section 4.2.2. All steroid-containing samples were subsequently analysed using UPC^2^-MS/MS, as previously described by our group [32]. Briefly, steroids were separated on an ACQUITY UPC^2^ system (Waters Corporation, Milford, CN, USA) with an ethylene-bridged hybrid 2-ethylpyridine (BEH-2EP) column (3.0 × 100 mm, 1.7 µm) and a Van Guard pre-column (2.1 × 5 mm, 3.5 µm). The quantitative mass spectrometric detection was achieved with a Xevo TQ-S triple quadrupole mass spectrometer (Waters Corporation, Milford, CT, USA) in multiple reaction monitoring (MRM) mode using the positive electrospray ionisation (ESI+) mode. All data were collected, analysed and quantified using the MassLynx 4.1 software package.

### 4.3. Receptor Assays

Confluent CV1-ARluc and CV1-luc cells were propagated into 48-well plates at a cell-count of 5 × 10^4^ cells/mL (500 µL/well) and were subsequently incubated for 24 h. For the PRA and PRB assays, CV1-luc cells were transiently transfected prior to ligand addition. Transfections were completed as per the manufacturer’s instructions, whereby transfection complexes (50 µL/well) were made, which included cDNA for PRA, PRB or pCIneo (0.01 mg/mL), the XtremeGene^®^ HP transfection reagent (3 µL reagent/µg cDNA) and non-supplemented DMEM/F12. The transfection complex was added to media and cells in a drop-wise manner prior to being incubated for an additional 24 h. Steroid addition took place post-incubation for all assays and required aspiration of the media and replacement with the treatment media. The treatment media were fresh DMEM/F12 supplemented with charcoal-stripped FBS free of endogenous steroids (10%), penicillin-streptomycin (1%) and the relative steroid treatment. Briefly, charcoal-stripped FBS was made by the preparation and incubation of dextran coated charcoal (DCC) (0.25 M of sucrose, 1.5 mM of MgCl_2_, 10 mM of HEPES, 0.25% Norit A charcoal and 0.0025% dextran T70) overnight. Following incubation, the DCC was isolated from the supernatant by means of centrifugation and was further incubated with the FBS (253 mg of DCC/100 mL of serum) for an additional 12 h period. Thereafter, the FBS was filtered and analysed for endogenous steroids and was stored at −20 ℃ prior to use. Treatments further entailed steroids assayed for both agonistic and antagonistic activity (Table 1). A steroid stock solution was made (1 mg/mL) for each treatment from which additional stocks were developed by serial dilution (0.1 mg/mL, 0.01 mg/mL and 0.001 mg/mL). These stocks (indicated in brackets) were utilised for the addition of ligands at various concentrations, namely, 10 nM (0.001 mg/mL of the stock), 100 nM (0.01 ng/mL of the stock) and 1 × 10^3^ nM (0.1 mg/mL of the stock) for agonism, while antagonism was assayed at the same concentrations but with the addition of 1 × 10^4^ nM (1 mg/mL of the stock). The treatment was added to both CV1-luc and CV1-ARluc and to transiently transfected cells, where untransfected CV1-luc cells served as the negative control in the case of the AR assays while the PRA and PRB assays utilised CV1-luc cells expressing pCIneo as the negative control. Moreover, additional cells were treated with ethanol only to determine basal activity to which all experimental results could be normalised. All plates were subsequently incubated for 24 h, after which samples (25 µL) were collected and transferred to 96-well white, opaque bottom plates. A stock solution of coelenterazine (1 mg/mL) was prepared and diluted 1:100 with a buffer (50 mM of TRIS and 150 mM of NaCl), and luminescence was measured by Tecan Spark 10 M, as per the manufacturer’s instruction. 

### 4.4. Statistical Analyses

GraphPad Prism 9 was used for all statistical analyses. All experiments were performed in triplicate, and results are represented as mean ± SEM. The 11βHSD conversion assay data were analysed for statistical significance with an unpaired *t*-test comparing substrate added vs. substrate remaining. Moreover, data obtained from the receptor assays were analysed using two-way ANOVA followed by a Fisher’s LSD multiple comparisons test to determine the significance of the results in comparison to natural ligands of the relevant receptors. Statistically significant differences are shown as *, **, *** or **** which represent *p* < 0.05, *p* < 0.005, *p* < 0.001 and *p* < 0.0001, respectively, while non-significant differences are indicated by §.

## Figures and Tables

**Table 1:** Steroids assayed for agonist and/or antagonist activity towards the androgen receptor and progesterone sub-type A and B receptors.

Androgens		Progestogens	
A4	androstenedione	P4	progesterone
11OHA4	11β-hydroxyandrostenedione	11αOHP4	11α-hydroxyprogesterone
11KA4	11-ketoandrostenedione	11βOHP4	11β-hydroxyprogesterone
T	testosterone	11KP4	11-ketoprogesterone
11OHT	11β-hydroxytestosterone	11αOHDHP4	11α-hydroxydihydroprogesterone
11KT	11-ketotestosterone	11βOHDHP4	11β-hydroxydihydroprogesterone
5αdione	5α-androstanedione	11KDHP4	11-ketodihydroprogesterone
11OH5αdione	5α-androstan-11β-ol-3,17-dione	3,11-diOHDHP4	5α-pregnan-3α,11β-diol-20-one
11K5αdione	5α-androstane-3,11,17-trione	alfaxalone	5α-pregnan-3α-ol-11,20-dione
DHT	dihydrotestosterone	17OHP4	17α-hydroxyprogesterone
11OHDHT	11β-hydroxydihydrotestosterone	21dF	21-deoxycortisol
11KDHT	11-ketodihydrotestosterone	21dE	21-deoxycortisone
AST	androsterone	11OHPdione	5α-pregnan-11β,17α-diol-3,20-dione
11OHAST	11β-hydroxyandrosterone	11KPdione	5α-pregnan-17α-ol-3,11,20-trione
11KAST	11-ketoandrosterone	11OHPdiol	5α-pregnan-3α,11β,17α-triol-20-one
11OH3αdiol	11β-hydroxyandrostanediol	11KPdiol	5α-pregnan-3α,17α-diol-11,20-dione
11K3αdiol	11-ketoandrostanediol		

**Table 2:** **A.** AR agonism exhibited by C_19_ and C11-oxy C_19_ steroids. Activity is shown as a percentage of the highest response of the canonical ligand (shaded row). Results are presented as mean ± SEM (n = 3). **B.** Antagonism of the AR by C_19_ and C_21_ steroids in the presence of 10 nM of T. Activity is shown as a percentage of the highest response of the canonical ligand alone (shaded steroid). Results are presented as mean ± SEM (n = 3).

(A)									
	10 nM			100 nM			1 µM		
Steroid	% Induction	±SEM	Statistical Significance	% Induction	±SEM	Statistical Significance	% Induction	±SEM	Statistical Significance
T	3.18	1.29	-	35.1	9.66	-	100	16.6	-
DHT	30.4	9.92	*	141	35.7	****	161	22.4	****
11KT	1.81	0.71	§	15.0	4.52	§	148	25.6	****
11KDHT	6.59	1.2	§	78.0	11.6	***	191	45.9	****
11OHDHT	1.63	0.49	§	10.7	4.53	*	153	49.1	****
**(B)**									
	**1 µM**					**10 µM**			
**Steroid**	**% Induction**		**±SEM**	**Statistical** **Significance**		**% Induction**	**±SEM**	**Statistical** **Significance**	
T	100		2.86	-		100	2.86	-	
Bicalutamide	71.0		3.95	§		11.3	1.55	****	
11OHA4	103		11.5	§		62.1	7.53	*	
11OH5αdione	103		15.4	§		68.5	8.35	*	
11αOHP4	94.7		13.2	§		23.3	0.15	****	
11αOHDHP4	90.9		3.41	§		24.2	10.7	****	
11βOHP4	57.5		8.01	**		7.60	1.5	****	
11βOHDHP4	76.2		11.6	§		18.4	0.88	****	
11KP4	76.3		15.2	§		25.7	4.25	****	
11KDHP4	75.1		22	§		34.9	9.73	****	
21dE	110		11.3	§		68.8	4	*	
11KPdione	92.7		14.7	§		38.8	6.6	****	
P4	22.3		5.11	****		9.93	3.29	****	
17OHP4	72.2		13.1	§		10.8	1.15	****	

§, not significant; *, <0.05; **, <0.005; ***, < 0.001; ****, <0.0001.

**Table 3:** PRA agonism exhibited by C_21_ and C11-oxy C_21_ steroids. Activity is shown as a percentage of the highest response of the canonical ligand (shaded steroid). Results are presented as mean ± SEM (n = 3).

	10 nM			100 nM			1 µM		
Steroid	% Induction	±SEM	Statistical Significance	% Induction	±SEM	Statistical Significance	% Induction	±SEM	Statistical Significance
P4	83.9	12.7	-	93.9	9.22	-	100	12.4	-
17OHP4	47.5	2.04	****	50.5	1.52	****	55.4	2.22	****
11βOHP4	62.3	10.6	**	68.7	7.87	***	91.6	12.6	§
11βOHDHP4	48.6	3.29	****	51.0	2.54	****	73.4	12.1	***
11αOHP4	49.9	4.69	****	55.4	5.42	****	67.4	8.52	****

§, not significant; **, <0.005; ***, <0.001; ****, <0.0001.

**Table 4:** **A.** PRB agonism exhibited by the C11-oxy C_19_ steroids, C_21_ steroids and C11-oxy C_21_ steroids. Activity is shown as a percentage of the highest response of the canonical ligand (shaded steroid). Results are presented as mean ± SEM (n = 3). **B.** Antagonism of the PRB by C_19_ and C_21_ steroids in the presence of 10 nM of P4. Activity is shown as a percentage of the highest response of the canonical ligand alone (shaded steroid). Results are presented as mean ± SEM (n = 3).

(A)												
	10 nM				100 nM				1 µM			
Steroid	% Induction	±SEM	Statistical Significance		% Induction	±SEM	Statistical Significance		% Induction	±SEM	Statistical Significance	
P4	43.5	2.42	-		87.4	14.2	-		100	23.9	-	
17OHP4	13.1	0.90	***		15.1	1.15	****		35.4	5.04	****	
11βOHP4	25.0	5.79	§		113	10.5	**		124	16.2	*	
11βOHDHP4	13.9	0.72	**		21.7	4.83	****		80.7	22.2	*	
11KP4	13.8	0.37	**		14.9	0.96	****		31.4	5.55	****	
11αOHP4	12.7	1.32	***		17.2	3.03	****		45.4	6.88	****	
11K3αdiol	41.2	27.9	§		45.3	32.9	****		57.4	19.6	****	
**(B)**												
	**10 nM**			**100 nM**			**1 µM**			**10 µM**		
**Steroid**	**% Induction**	**±SEM**	**Statistical Significance**	**% Induction**	**±SEM**	**Statistical Significance**	**% Induction**	**±SEM**	**Statistical Significance**	**% Induction**	**±SEM**	**Statistical Significance**
P4	100	2.9	-	100	2.9	-	100	2.9	-	100	2.9	-
RU486	99.3	2.21	§	11.7	0.75	****	10.5	0.39	****	9.64	0.21	****
A4	51.9	8.81	****	44.7	5.04	****	47.7	7.64	****	29.7	5.11	****
T	82.9	18	§	64.5	8.95	**	48.4	8.92	****	34.9	1.02	****
DHT	72.1	1.19	*	65.5	4.75	**	55.8	3.36	***	34.6	0.86	****
5αdione	74.7	4.02	*	56.5	2.13	***	68.9	4.63	**	38.0	5.47	****
11OHA4	51.2	8.18	****	48.5	6.87	****	56.6	10.3	***	30.6	1.29	****
11OHT	62.5	2.51	**	68.5	6.68	**	66.0	8.73	**	48.4	5.92	****
11KT	69.1	14.4	**	78.2	16.7	§	61.4	8.77	**	35.6	1.83	****
11KDHT	85.9	10.9	§	85.0	11.7	§	74.4	5	*	45.0	2.5	****

§, not significant; *, <0.05; **, <0.005; ***, <0.001; ****, <0.0001.

## Data Availability

Data are contained within the article and Supplementary Materials.

## Supplementary Materials

The following supporting information can be downloaded at: https://www.mdpi.com/article/10.3390/ijms25010101/s1.
