# Supplementary material for: C11-hydroxy and C11-oxo C19 and C21 Steroids: Pre-Receptor Regulation and Interaction with Androgen and Progesterone Steroid Receptors
Source: Int J Mol Sci. 2023 Dec 20;25(1):101. doi: 10.3390/ijms25010101 (PMC10778819; doi:10.3390/ijms25010101)
Supplement: Supplementary file 1 [file ijms-25-00101-s001.zip › ijms-2736103-supplementary.pdf]

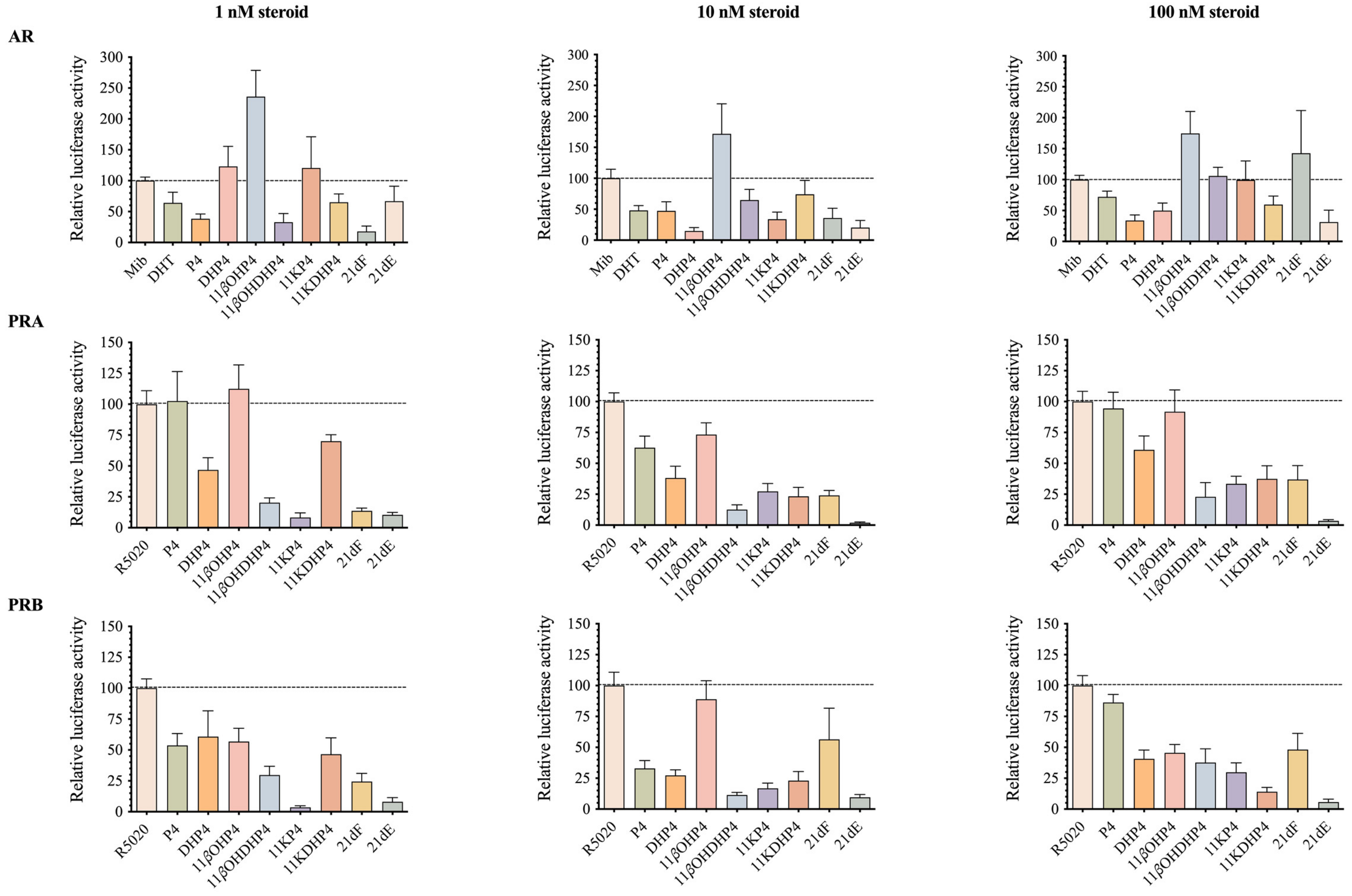

Figure S1. Transactivation of C11-oxy C_{21} steroids towards the human androgen receptor (AR), progesterone receptor A (PRA), progesterone receptor B (PRB). Steroids were assayed with 1, 10 and 100 nM steroid substrate. Data represent three independent experiments performed in quadruplicate, except for

11β-hydroxydihydroprogesterone (11βOHDHP4) which is representative of one independent experiment. Data was normalised to protein. Agonist activity depicted relative to mibolerone (Mib) and R5020.

Luciferase reporter promoter assays in HEK293 cells (Figure S1)

HEK293 cells were cultured in DMEM, pH 7.0, supplemented with 1.5 g/L $NaHCO_3$, FBS (10%) and 1% Pen-Strep and upon confluency were replated into 100 mm CellBIND dishes at 2 x 10^5 live cells/mL. Cells were co-transfected with the plasmids expressing the AR and PR isoforms, 0.9 μg, and the response element (9 μg), and incubated for 24 h. The transfected cells were replated into ViewPlate-96 at 5 x 10^4 live cells/mL using phenol red-free DMEM media supplemented with 10% dextran-stripped FBS and 1 % Pen-Strep. After 24 h, the media was aspirated and cells treated with the steroid substrate (1, 10 and 100 nM, and 1 μM prepared in unsupplemented phenol red-free DMEM) and incubated for 24 h. The cells were treated with a vehicle control (ethanol, 0.1%), positive controls mibolerone and dihydrotestosterone for the androgen receptor, R5020 and progesterone for the progesterone receptor isoforms, and C11-oxy C_{21} steroids. The luciferase reporter promoter assay and subsequent protein determination to which luciferase activity was normalised to was done as stipulated by the manufacturer.

Table S1. Antagonism of the androgen receptor (AR) by C_{19} and C_{21} steroids, 10 nM and 100 nM, in the presence of 10 nM T. Activity is shown as a percentage of the highest response of the canonical ligand alone (shaded steroid). §, non-significant/P > 0.05.

**Concentration:**	**10nM**			**100nM**		
**Steroid**	**% Induction**	**±SEM**	**Statistical Significance**	**% Induction**	**±SEM**	**Statistical Significance**
T	100	2.86	-	100	2.86	-
Bicalutamide	117	7.97	§	98.2	5.61	§
11OHA4	109	18.2	§	120	20.5	§
11OH5αdione	128	7.76	§	111	16.1	§
P4	103	19.2	§	73.9	18.9	§
11βOHP4	102	4.73	§	88.9	8.19	§
17OHP4	116	3.25	§	106	8	§
11βOHDHP4	85.7	5.45	§	95.1	2.84	§
11αOHP4	124	13.1	§	128	18.4	§
11αOHDHP4	108	10.4	§	107	1.01	§
11KP4	94.1	8.2	§	83.7	13.5	§
11KDHP4	89.9	7.82	§	93.2	11.2	§
11KPdione	101	20	§	103	12.6	§
21dE	106	6.94	§	109	5.9	§

Table S2. Antagonism of the progesterone receptor A (PRA) by the C_{19} steroids, C11-oxy C_{19} steroids, C_{21} steroids and C11-oxy C_{21} steroids in the presence of 10 nM P4. Activity is shown as a percentage of the highest response of the canonical ligand alone (shaded steroid). **, P < 0.005.

**Concentration:**	**10nM**			**100nM**			**1µM**			**10µM**		
**Steroid**	**% Induction**	**±SEM**	**Statistical Significance**	**% Induction**	**±SEM**	**Statistical Significance**	**% Induction**	**±SEM**	**Statistical Significance**	**% Induction**	**±SEM**	**Statistical Significance**
P4	100	3.2	-	100	3.2	-	100	3.2	-	100	3.2	-
RU486	50.8	2.52	**	50.9	3.49	**	51.9	2.65	**	42.2	3.69	**
